# Concha bullosa mucocele with orbital invasion and secondary frontal sinusitis: a case report

**DOI:** 10.1186/1756-0500-6-501

**Published:** 2013-12-03

**Authors:** Jung-Hoon Lee, Sung-Lyong Hong, Hwan-Jung Roh, Kyu-Sup Cho

**Affiliations:** 1 Department of Otorhinolaryngology and Biomedical Research Institute, Pusan National University School of Medicine, Busan, South Korea; 2 Department of Otorhinolaryngology, Pusan National University School of Medicine Yangsan Hospital, Yangsan, South Korea

**Keywords:** Turbinates, Mucocele, Frontal sinusitis, Orbit, Complications

## Abstract

**Background:**

Although concha bullosa (CB) is the most common variants of the middle turbinate, mucocele of CB is uncommon. Furthermore, CB mucocele with orbital invasion and secondary frontal sinusitis has not been reported previously.

**Case presentation:**

A 42-year-old Korean male presented with gradually progressive proptosis of right eye and right-sided frontal headache. He had previously undergone endoscopic sinus surgery (ESS) 15 and 9 years ago. The endoscopic examination showed an expansive, large middle turbinate with normal mucosa filled the majority of right nasal cavity and displaced the septum to the left. A computed tomography and magnetic resonance imaging showed a well demarcated cystic huge mass at right nasal cavity extending to ethmoid sinus and orbit. The mass caused a bony defect on the lamina papyracea and displaced medial rectus muscle and orbit laterally. Moreover, the right frontal and ethmoid sinus was totally opacified. This article reports orbital invasion and frontal sinusitis complicating a CB mucocele, which was successfully treated by endoscopic resection of the lateral wall of CB and frontal sinusotomy.

**Conclusions:**

This case illustrates that CB mucocele could develop to such a massive extent that it leads to orbital complication and secondary frontal sinusitis. Therefore, we consider this entity in the differential diagnosis of orbital complications and secondary sinusitis caused by intranasal mass.

## Background

A mucocele is an epithelium-lined cavity containing mucus which fills the paranasal sinus and is capable of expansion [1]. The majority of mucoceles are situated in the ethmoid and/or frontal sinuses [1,2]. If a mucocele becomes infected, it is referred to as a pyocele [3]. Although concha bullosa (CB) is the most common variants of the middle turbinate, mucocele or pyocele of CB has been rarely reported [2-5]. Furthermore, CB mucocele with orbital extension is much less and CB mucocele with orbital invasion and secondary frontal sinusitis has not been reported in the literature, to the best of our knowledge. Here, we describe this rare clinical presentation of orbit invasion and frontal sinusitis complicating massive mucocele of CB. This study was approved by the Institutional Review Board of Pusan National University Hospital.

## Case presentation

**Figure 1:**
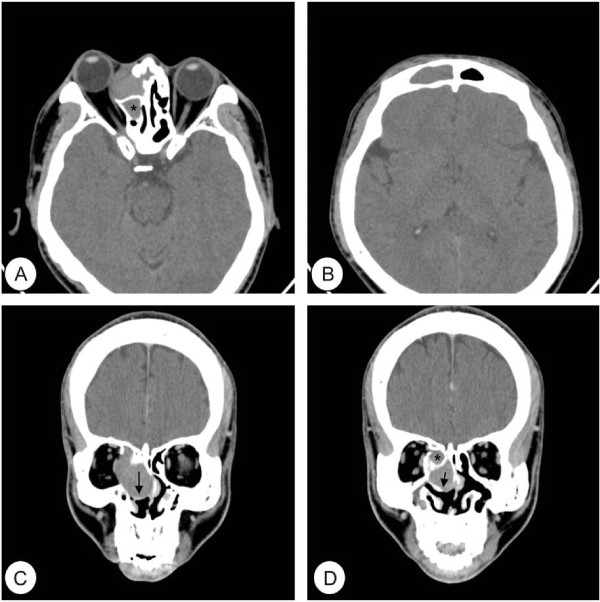
**Preoperative computed tomography (CT) images of the orbit.** Axial **(A, B)** and coronal **(C, D)** CT images show a well demarcated mass surrounded by a bony perimeter of the middle turbinate (arrow) at right nasal cavity. Medial orbital wall is destructed and nasal septum is deviated to the left side. The medial rectus muscle and orbit is displaced laterally. Total opacification is noted in right ethmoid sinus (asterisk) and frontal sinus.

**Figure 2:**
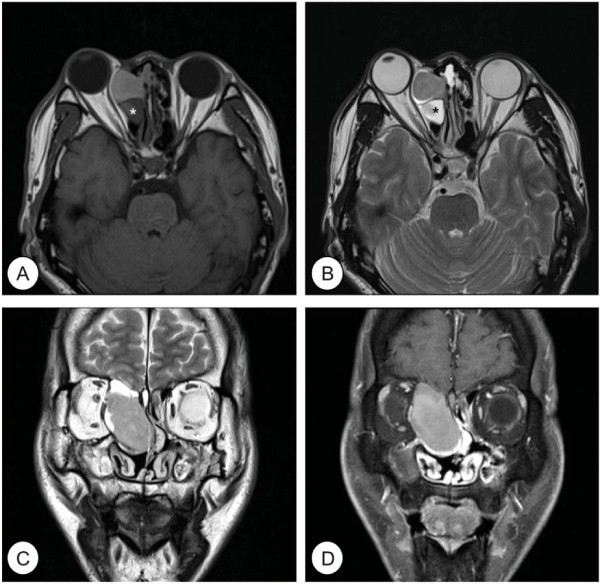
**Preoperative magnetic resonance (MR) images of the paranasal sinus.** MR images show a huge cystic mass displaying intermediate signal intensity on T1 axial **(A)**, T2 axial **(B)**, and T2 coronal images **(C)**. Post-contrast T1 mage **(D)** show no enhancement of the cystic mass. Right ethmoidal sinusitis is noted (asterisk).

A 42-year-old Korean male presented with gradually progressive proptosis of right eye for 2 years. He also complained of right-sided frontal headache for 1 week. He had previously undergone endoscopic sinus surgery (ESS) for chronic rhinosinusitis with nasal polyp 15 and 9 years ago. There were no other rhinological or ophthalmological symptoms. He had suffered from adult onset diabetes mellitus for 5 years and was well controlled on oral hypoglycemic agents. The endoscopic examination showed an expansive, large middle turbinate with normal mucosa filled the majority of right nasal cavity and displaced the septum to the left. Ophthalmologic examination revealed exophthalmos and globe was displaced antero-laterally. Ocular motion and visual acuity were normal. A computed tomography (CT) scan of the orbit showed a well demarcated huge mass at right nasal cavity extending to ethmoid sinus and orbit. The mass caused a bony defect on the lamina papyracea and displaced medial rectus muscle and orbit laterally. Moreover, the right frontal and ethmoid sinus was totally opacified (Figure 1). Magnetic resonance (MR) images of the paranasal sinus revealed a cystic mass displaying intermediate signal intensity on T1-weighted images (T1WIs) and T2WIs without enhancement. In addition, right frontal and ethmoid sinusitis was also observed (Figure 2). From these findings, the lesion was suspected to be a CB mucocele with orbital invasion and frontal sinusitis. Resection of the lateral and inferior walls of the right middle turbinate was performed under general anesthesia combined with a right frontal sinusotomy. The middle turbinate consisted of thick, dark brown, and inspissated material surrounded by a partially bony shell with mucosa at both inner and outer side, confirming the diagnosis of a mucocele in CB. No organism was seen on gram, AFB, and fungus stain and culture yielded no growth in the pus from CB. After frontal sinusotomy, pulsating pus like discharge and inflammation-induced edematous sinus mucosa were noted. An oral antibiotic treatment with Amoxicillin and Clavulanate was started for concurrent paranasal sinus infection, and planned to complete for 2 weeks. The patient’s symptoms quickly diminished postoperatively and a follow-up CT scan 9 month after surgery demonstrated complete resolution of CB mucocele and frontal sinusitis.

## Discussion

CB that is a pneumatization of the middle turbinate is one of the most common variations of the sinonasal anatomy [3]. Like other aerated cells, the CB possesses a mucociliary transport system [5], with the ostium connecting the air cell lumen to the frontal recess [2]. The obstruction of its ostium could lead to mucocele and even pyocele after infection of retained secretion. Different etiological factors may lead to such obstruction, including nasal polyps, surgery, trauma, and benign tumors [2]. In the present case, the previous ESS may have been relevant by causing local scarring with stenosis or obstruction of the drainage of the CB.

CB alone is usually asymptomatic. However, in the formation of a mucocele, CB may become expanded and occupy the surrounding structures with local bone destruction. Therefore, headache, orbital pain, exophthalmos, nasal discharge, postnasal drip, nasal obstruction, and anosmia are all possible symptoms of a patient with CB mucocele [5]. In our case, the patients had exophthalmos and frontal headache which are considered to be caused by extension of CB mucocele into the orbital space and acute frontal sinusitis secondary to the obstruction of osteomeatal complex by CB mucocele.

The diagnosis could be suspected from its characteristic radiologic findings. In this study, orbit CT image showed a prominent soft tissue mass well circumscribed by a bony framework of the CB. This is important differential diagnosis between CB and ethmoidal mucocele [5]. Moreover, MR images showed intermediate signal intensity on T1WIs and T2WIs, implying the CB is filled with higher protein content of mucocele.

The traditional treatment for CB mucocele is endoscopic surgery and there are four methods to manage surgically: lateral marsupialization, medial marsupialization, crushing, and transverse excision [6]. Generally, the choice of surgical access depends on the localization and extension of the mucocele. We performed the excision of lateral and inferior part of the CB to remove all pus easily and prevent recurrence. It is important to avoid excessive manipulation of the medial aspect of the CB because the medial lamella attaches to the skull base.

## Conclusion

This case illustrates that CB mucoceles could develop to such a massive extent that it leads to orbital complication and secondary frontal sinusitis. Therefore, we consider this case in the differential diagnosis of orbital complications and secondary sinusitis caused by intranasal mass. Resection of the lateral wall of the CB and frontal sinusotomy will be needed to properly treat this condition.

## Consent

Written informed consent was obtained from the patient for publication of this manuscript and accompanying images. A copy of the written consent is available for review by the Editor-in-Chief of this journal.

## Competing interests

The authors declare that they have no competing interests.

## Authors’ contributions

The author JHL has been involved in drafting the manuscript or revising it. SLH and HJR have obtained the data and figures, and drafted the manuscript and references. KSC has made the study design and concept, drafted the manuscript, and made critical review. All authors read and approved the final manuscript.
